# Identifying Neutrophil Extracellular Traps (NETs) in Blood Samples Using Peripheral Smear Autoanalyzers

**DOI:** 10.3390/life13030623

**Published:** 2023-02-23

**Authors:** Kateryna Fedorov, Mohammad Barouqa, David Yin, Margarita Kushnir, Henny H. Billett, Morayma Reyes Gil

**Affiliations:** 1 Division of Hematology, Albert Einstein College of Medicine, Bronx, NY 10467, USA; 2 Division of Hematology, Department of Oncology, Montefiore Medical Center, Albert Einstein School of Medicine, 3411 Wayne Ave, Ground Floor, Bronx, NY 10467, USA; 3 Department of Pathology, Albert Einstein College of Medicine, Bronx, NY 10467, USA; 4 Department of Internal Medicine, Montefiore Medical Center, Albert Einstein College of Medicine, Bronx, NY 10467, USA; 5 Medical Director Hemostasis and Thrombosis Laboratories Cleveland Clinic, Cleveland, OH 44195, USA

**Keywords:** neutrophil extracellular traps, peripheral blood, sepsis, infection, digital white blood cell differential

## Abstract

Neutrophil Extracellular Traps (NETs) are large neutrophil-derived structures composed of decondensed chromatin, cytosolic, and granule proteins. NETs play an important role in fighting infection, inflammation, thrombosis, and tumor progression processes, yet their fast and reliable identification has been challenging. Smudge cells (SCs) are a subcategory of white cells identified by CellaVision^®^, a hematology autoanalyzer routinely used in clinical practice that uses digital imaging to generate “manual” differentials of peripheral blood smears. We hypothesize that a proportion of cells identified in the SC category by CellaVision^®^ Hematology Autoanalyzers are actually NETs. We demonstrate that NET-like SCs are not present in normal blood samples, nor are they an artifact of smear preparation. NET-like SCs stain positive for neutrophil markers such as myeloperoxidase, leukocyte alkaline phosphatase, and neutrophil elastase. On flow cytometry, cells from samples with high percent NET-like SCs that are positive for surface DNA are also positive for CD45, myeloperoxidase and markers of neutrophil activation and CD66b. Samples with NET-like SCs have a strong side fluorescent (SFL) signal on the white count and nucleated red cells (WNR) scattergram, representing cells with high nucleic acid content. When compared to patients with low percent SCs, those with a high percentage of SCs have a significantly higher incidence of documented bacterial and viral infections. The current methodology of NET identification is time-consuming, complicated, and cumbersome. In this study, we present data supporting identification of NETs by CellaVision^®^, allowing for easy, fast, cost-effective, and high throughput identification of NETs that is available in real time and may serve as a positive marker for a bacterial or viral infections.

## 1. Introduction

Neutrophils are an integral part of the immune system’s first line of defense against foreign organisms. Neutrophils’ antimicrobial properties encompass three processes: phagocytosis, degranulation, and release of neutrophil extracellular traps (NETs) [1,2,3]. NETs are large cell-derived structures composed of decondensed chromatin, cytosolic and granule proteins. They function by trapping, neutralizing, killing, and preventing dissemination of infectious pathogens such as bacteria, viruses, fungi, and parasites [4,5,6,7]. Sterile inflammatory processes have also been associated with increased NET production in which case they have been implicated in pathologic amplification of inflammation, cytokine release, and tissue damage. In addition to their antimicrobial functions, NETs are thought to play an important role in pathogenesis of autoimmunity, vaso-occlusion, thrombosis, tumor capture, and spread [2,8,9,10].

Since their initial discovery in 2004, the scientific community has been working on finding reliable in-vivo NET identification methods [5]. Currently, visual identification of NETs relies on the visualization of DNA, histones, myeloperoxidase, and neutrophil elastase, using advanced immunohistochemical staining and microscopic techniques [5,9,11,12]. Alternatively, cell-free DNA, MPO-DNA complexes, citrullinated histones C3, neutrophil elastase, and cathelicidin antimicrobial peptides are used as surrogate markers for NETosis [13,14,15,16,17,18]. Neutrophil activation marker CD66b has been described as positively correlating with side-fluorescent scatter (SFL) generated by the Sysmex system [18,19], with several reports suggesting that NETs are identified with Sysmex’ SFL scatters [18,20]. Such techniques are time-consuming, complicated, and cumbersome, hindering expansion into clinical practice. Currently, there are no readily available tools for the rapid clinical identification of NETs in patients.

Sysmex is an automated Hematology System routinely used in clinical laboratories for quantification of cells within peripheral blood and body fluid samples. Sysmex’s White Count and Nucleated Red Cell channel (WNR) uses fluorescent flow cytometry employing polymethine dye for nucleic acids and a cell-specific lyse to capture side fluorescence (SFL) measuring cell’s nucleic acid content and complexity and forward scatter (FSC) assessing cell size [21]. Automated image analyzers such as the CellaVision^®^ Hematology Autoanalyzer are frequently used together with Sysmex. Blood samples flagged by Sysmex as abnormal are routed for peripheral blood smear generation and further analysis by CellaVision^®^. CellaVision^®^ takes images of the cells on the slide and categorizes them based on morphology, generating a “manual” differential. Digital images of white blood cells (WBC) on the blood smear can subsequently be accessed and reviewed by clinical personnel. One of CellaVision^®^’s WBC categories is termed smudge cells (SC). It contains cellular entities that have lost their structural integrity and cannot be morphologically classified any further. Classically, on the peripheral blood smear, the smudge cells have been thought to represent degenerated lymphocytes (DL), which are abundant in blood smears of patients with chronic lymphocytic leukemia (CLL) [22]. Such smudge cells are a result of breakdown of fragile leukemic lymphocytes and, therefore, represent an artifact of smear preparation [22]. We observed that a proportion of cell derived entities categorized as smudge cells by CellaVision^®^ morphologically resembled NETs. Sysmex WNR scattergrams that corresponded to blood samples with abundant SC have an increased population of unique, cellular entities that are comparable to WBC in size (FSC), but have high nucleic acid content (SFL).

We hypothesize that, in addition to the classic degenerated lymphocytes, a proportion of SC, as identified by the CellaVision^®^ Hematology Autoanalyzer, are actually NETs. Here, we present supporting albeit preliminary data showing that NET-like SC are not present in normal blood samples and are not specimen handling artifacts, display neutrophil and NET specific markers on immunohistochemistry and flow cytometry, and generate unique patterns on Sysmex WNR scattergrams comparable to those seen in scattergrams of samples induced with NET trigger reagents. Additionally, we show that increased percent SC in the samples of hospitalized patients correlates positively with infection rates.

## 2. Materials and Methods

*CellaVision^®^ peripheral smear images and Sysmex WNR scattergram analysis.* Clinical laboratories at Montefiore Medical Center use Sysmex CBC analyzers (XN 9000) and CellaVision^®^ (v6.0.3) for routine analysis of EDTA-whole blood for CBC, peripheral blood smear generation using Wright Giemsa stain, and acquisition of digital images of these smears to classify white blood cells and generate a manual differential count. The smudge cell category reported by CellaVision^®^ includes the classic degenerated lymphocytes, but also cellular entities that resemble NETs. We initially identified NET-like SC using a set of morphologic characteristics: lack of discernible plasma membrane, no intact cytoplasm, dispersed granules, decondensed and congested nuclei, and polarized chromatin projections. To investigate whether such NET-like SC are detectable by Sysmex’s WNR (White Count and Nucleated Red Blood Cells) scattergrams corresponding to samples, we assessed the area of moderate FSC and high SFL, as it is known that NETs are similar in size to WBC but have a higher nucleic acid and granulation. To quantify the fluorescence signal in the WBC and NET area of WNR scattergram we used ImageJ^®^ software (v1.53a). 

*Flow cytometry characterization of NETs.* To further characterize NET-like SC as of neutrophil origin, we selected samples with ≤5% SC, ≥20% with majority SC morphologically characterized as NETs, and ≥20% with majority degenerated lymphocytes. To maintain cell integrity, centrifugation and cell permeabilization was avoided. Antibodies to SYTOX green, CD45, MPO, Neutrophil Elastase (NE), and CD66b were added to the buffy coats of gravity-separated EDTA whole blood samples. All antibodies were purchased from Invitrogen, Waltham MA and were used according to manufacturer recommendations. Gating was done using the Sytox positive cell gate, since only the SC should have surface DNA and be in this category, followed by gates for CD45, MPO and CD66b. 

*Effects of specimen handling and slide preparation (Incubation time, smearing angle, and pressure).* To determine whether NET-like SC were a product of sample handling and slide preparation, samples with no detectable morphological “NETs” or smudge cells as analyzed by the CellaVision^®^ underwent different slide preparation techniques. Five samples were incubated for 2, 6, 8, 10 and 24 h at 37 °C. Peripheral blood smears of these samples were processed through the CellaVision^®^ for analysis. Different angles (60°, 30°, 15°, 0°, and standard 45°) and different pressures (as hard as possible without breaking the slide and soft capillary pressure) were used in the preparation of manual slides. Smearing was performed using both push and pull techniques. As analysis of manually prepared slides by CellaVision^®^ is not possible, morphological NET counts were performed by microscopic examination of the slides by two different, blinded pathologists. 

*In vitro NET formation*. EDTA whole blood specimens without SC reported on CellaVision^®^ were then incubated with 100 nM phorbol 12-myristate 13-acetate (PMA), 100 μg/mL lipopolysaccharide (LPS), and 5 μM Ionomycin at 37 °C for 2, 6, 8, 10, and 24 h before re-processing through CellaVision^®^. All reagents were purchased from Sigma-Aldrich, St Louis, MO, USA. 

*Immunofluorescent staining for NET markers.* To ensure viable cell counts, 50 uL of Sytox green (Invitrogen, Waltham, MA, USA) was added directly to the EDTA whole blood tube prior to smear processing. For all other stains, blood smears were dried and permeabilized with 4% paraformaldehyde (PFA) in phosphate buffered saline (PBS) for 10 min and washed with PBS (both reagents purchased from Sigma-Aldrich, St. Louis, MO, USA). Smears were then incubated with anti-histone H3 (citrulline R2 + R8 + R17; Ab5103, from Abcam, Cambridge, UK) at 1:100 (in PBS + 1% bis(trimethylsilyl)acetamide (BSA)) followed by Alexa Fluoro 488 goat anti-rabbit IgG (purchased from Abcam, Cambridge, UK) at a 1:1000 dilution for 1 h. Immunohistochemistry staining for leukocyte alkaline phosphatase (LAP) activity and LAP scoring was performed as per manufacturer recommendations (Sigma-Aldrich, St. Louis, MO, USA). For myeloperoxidase (MPO) and neutrophil elastase staining, smears were stained with directly conjugated Alexa-Flouro488 MPO (Abcam, Cambridge, UK) at 1:1000 dilution for 1 h. 

*Correlating %SC in EDTA whole blood with presence of infections*. The CellaVision^®^ database was reviewed to randomly identify specimens with ≤5% and ≥20% SC between March 2018 to February 2020. After IRB approval, the corresponding electronic medical records were reviewed to collect data on demographics, %SC, WBC count, and presence of infections within 10 days of specimen collection (either confirmed by microbiology or documented as such by medical provider). Fluorescence signal of suspected NET area on Sysmex WNR scattergrams was quantified using ImageJ software (v1.53a).

*Statistical Analysis.* Statistical analyses of averages were performed using means and standard deviations or IQR for normal and non-normal distribution of data respectively. *p* values were calculated with Kruskal–Wallis test for continuous variable and Chi-Squared (or Fisher’s as needed) test for categorical variables using the R Studio^®^ software (v1.4.1717). Significance was denoted by a two tailed α = 0.05.

## 3. Results

### 3.1. Smudge Cells Are Not Increased in Normal Samples

To establish SC reference range for normal samples, 46 consecutive CBC specimens that were not flagged by Sysmex as abnormal were identified and manually routed to CellaVision^®^ for analysis. The median %SC on these normal samples was 4.3 [2.6; 4.3].

### 3.2. The CellaVision^®^ SC Category Contains Two Distinct Entities: Degenerated Lymphocytes and NET-like SC

**Figure 1:**
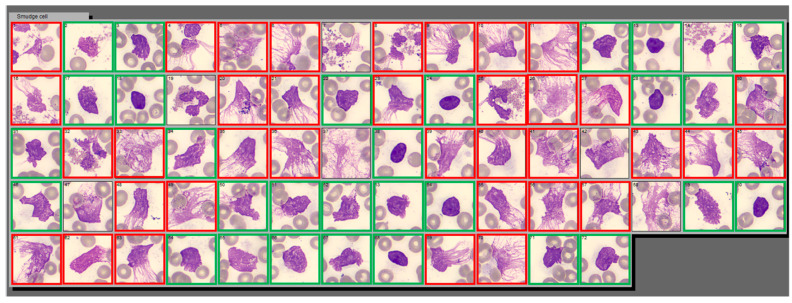
Example of Smudge Cell category in CellaVision^®^ containing two distinct populations of cell derived entities: NET-like and DL-like smudge cells. This is a screenshot of Smudge Cell category from Cellavision^®^ illustrating examples morphological differences between degenerating lymphocytes (green square) and NET-like smudge cells (red square). NET-like smudge cells are characterized by lack of discernible plasma membrane, no intact cytoplasm, dispersed granules, and polarized chromatin projections that resemble spider webs. Cellular entities that we were not able to classify as DL or NET-like were left without outline. Images are captured with a 100× magnification lens.

A proportion of cells in the SC category resembled classic degenerated lymphocytes (DL)—cellular remnants that did not retain any structural components, while other cells morphologically resembled NETs. On CellaVision^®^ NET-like SC appear as cell remnants with no discernible plasma membrane, no intact cytoplasm, dispersed granules, decondensed and congested nuclei, and polarized chromatin projections that resemble spider nets (Figure 1).

### 3.3. Sysmex WNR Scattergram Can Detect Differences in Nucleic Acid Content within WBC

**Figure 2:**
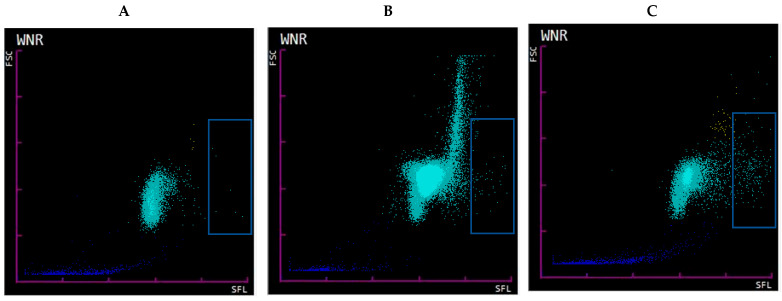
**Sysmex WNR scattergram.** WNR scattergram of EDTA-whole blood samples of hospitalized patients (**A**). Sample with ≤5% SC showing low signal in the high SFL area (blue box representing suspected NET area). (**B**). Sample with ≥20% majority DL-like SC with wide range of FSC and low-to-intermediate signal in the suspected NET area/high SFL. Such pattern is seen in samples with marked lymphocytosis secondary to CLL, high SFL signal is absent as fragile lymphocytes turn into smudge cells during smearing but stay intact during flow cytometry (**C**). Sample with ≥20% SC containing majority NET-like SC with FSC (size) within expected range for WBC but high SFL (nucleic acid content).

To further differentiate between DL-like and NET-like SC we evaluated samples with ≤5% SC, ≥20% SC with majority morphologic NET-like SC, and ≥20% SC majority with majority morphologic DL. WNR scattergram of samples with ≤5% SC had low-to-no signal at medium FSC (size) and high SFL (nucleic acid) areas (Figure 2, panel A). Samples with ≥20% DL-like SC had a distinct pattern with a strong and broad signal at higher-than-expected FSC, yet a low-to-intermediate signal in the high SFL region (compared to the samples with ≥20% NET-like SC) (Figure 2, panel B). This pattern was characteristic to samples of patients with lymphocytosis, mainly secondary to CLL. This pattern has been described by others as prolymphocytic cell clusters that form an “inverted comma” with high FSC in the WNR scattergram in chronic lymphoproliferative disorders [23]. Scattergrams of samples with ≥20% majority NET-like SC had visibly higher SFL signals within the expected FSC range for WBC (Figure 2, panel C). This further confirms that the SC category does contain two separate populations of cells: one with cells with increased nucleic acid contents and sizes comparable to WBC—likely representing NETs—and the second with cells without increased nucleic acid contents but with likely increased clumping that is represented by a large size range—likely representing increased numbers of fragile lymphocytes turning into smudge cells during smearing (hence ≥20% SC) but staying intact during flow cytometry.

### 3.4. Identification by Immunohistochemistry: Samples with Majority NETs-like SC Stain Positively with for LAP, MPO, and NE

**Figure 3:**
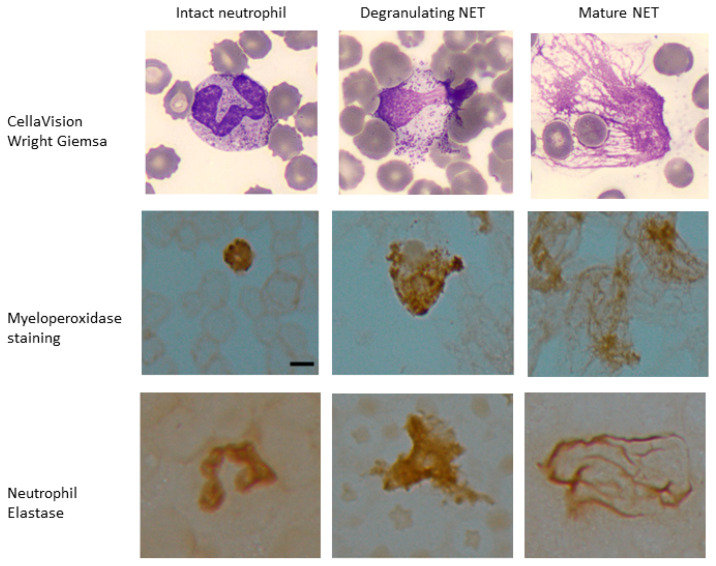
**Myeloperoxidase and neutrophil elastase staining of samples with high LAP score.** Peripheral blood samples with ≥20% NET-like smudge cells were stained and scored for leukocyte alkaline phosphatase (LAP). These samples also stained positive for neutrophil specific markers myeloperoxidase (MPO) and neutrophil elastase (NE) in intact neutrophils as well as degranulating neutrophils with features of nuclear decondensation and NET-like cells with web-like projections expressing myeloperoxidase and neutrophil elastase. Scale bar = 10 μm.

To further confirm our ability to identify NETs within the SC category, we assessed the cell of origin for NET-like and DL-like SC in samples with >20% SC. Twenty EDTA whole blood samples with >20% SC identified on CellaVision^®^, 10 with mostly NET-like SC and 10 with mostly DL-like SC, were selected for staining with LAP and blinded scoring. The samples with majority NET-like SC had LAP score of 172.1 and those with DL-like SC had a score of 103.3, *p* < 0.00087. Slides from patients with documented SC LAP scores were stained for MPO and NE; the NET-like SC cases stained strongly for MPO and NE (Figure 3).

### 3.5. Identification by Flow Cytometry: Samples with Majority NET-like SC Contain Surface MPO and CD66b

**Figure 4:**
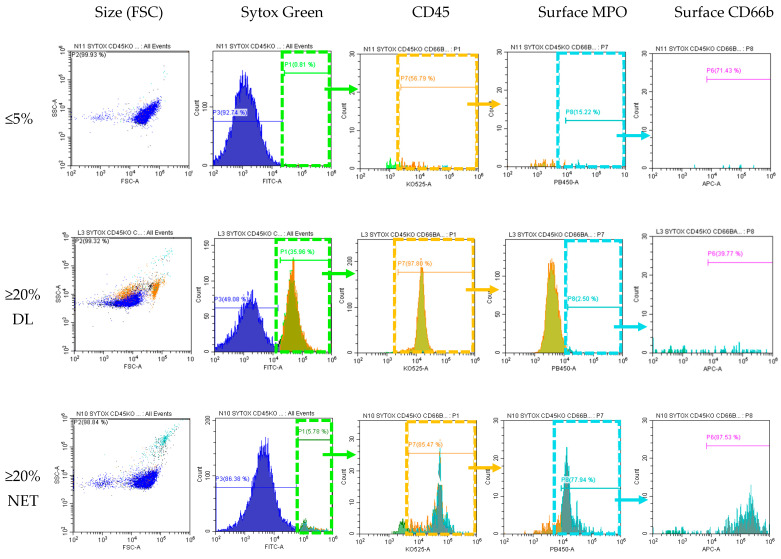
**Flow cytometry to differentiate NETs vs. Degenerated Lymphocytes.** Soft spin buffy coat samples were incubated unpermeabilized with antibodies for Sytox (surface DNA dye), CD45 (WBC marker K0525), surface MPO (PB450), surface CD66b (APC, marker for activated neutrophils). Sytox green positive cells were serially gated for CD45, then MPO, and then CD66b. Cells from sample with ≤5% SC did not express surface DNA and thus were negative for all subsequent gating. Cells from samples ≥20%SC with morphologically identified majority degenerated lymphocytes stain positive for Sytox+ and CD45+ but negative for MPO- and CD66b- (this population was back-gated and highlighted as orange in the SSC vs. FSC dot plot). Cells from samples ≥20%SC morphologically identified as majority NETs stain positive for all surface markers: Sytox+/CD45+/MPO+/CD66b+ (this population was back-gated and highlighted as cyan in the SSC vs. FSC dot plot). Note the difference in size and granulation between DL and NET-like SC.

To confirm the two groups within SC category that we identified using routinely available WNR scattergram and morphologic identification, we used flow cytometry to further characterize our populations. We used surface DNA (Sytox), CD45, MPO, and CD66b staining to identify SC and SC subtypes. Samples with ≤5% SC by CellaVision^®^ were usually negative by Sytox green while samples with ≥20% SC were positive. Sytox green positive cells were also positive for CD45, affirming that SC are of WBC origin. Surface MPO and CD66b within this Sytox+, CD45+ population was positive only in those samples with high NET-like SC but not in samples with high DL-like SC. This again affirms that the SC category contains both cells of neutrophil (Sytox green+/CD45+/MPO+/CD66b+) and lymphocyte origin (Sytox green+/CD45+/MPO-/CD66b−). This is demonstrated in Figure 4.

### 3.6. NET-like SC Are Not an Artifact of Specimen Handing

**Figure 5:**
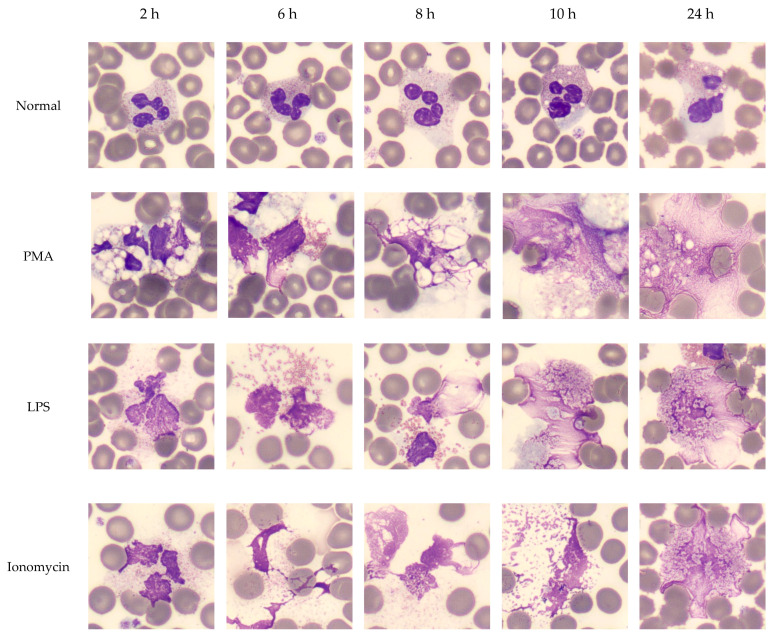
**Effect of time on NET formation in EDTA-whole blood samples.** NET formation was induced with classic triggers (PMA, LPS, Ionomycin) in EDTA whole blood from normal donors. Smears were prepared at 2, 6, 8, 10 and 24 h. WBC manual differential was performed by CellaVision^®^ to capture NETs at different stages within the smudge cell population. NET formation followed a canonical order of morphological changes: vacuolation, nuclear decondensation, degranulation, chromatin ejection and protrusions. Images are captured with a 100× magnification lens.

Compared to the standard smear preparation technique, neither larger or smaller angles nor increased smearing pressure resulted in greater numbers of NET-like SC (*p* = 0.14 for angle and *p* = 0.07 for pressure). When subjected to prolonged incubation times, EDTA-whole blood specimens did not develop increased number of NET-like SC (*p* = 0.09), although some vacuolation and expansion of WBC cytoplasm was observed (Figure 5, top row).

### 3.7. Induced NETs Have the Same Appearance as NET-like SC on CellaVision^®^ and Produce the Same Signal on WNR Scattergram

**Figure 6:**
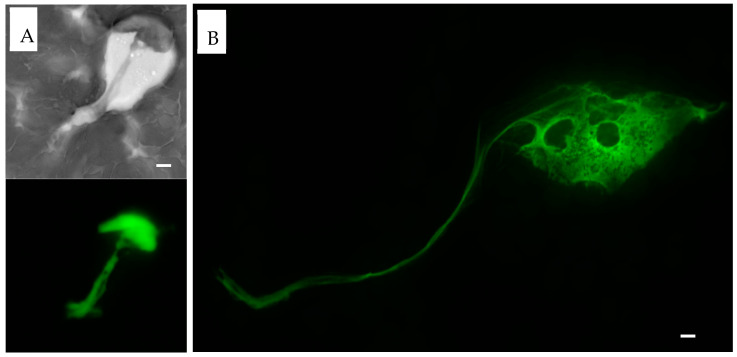
**Sytox green staining of PMA induced NETs.** EDTA-whole blood samples stimulated with PMA stained with Sytox green dye. (**A**). At 2 h, NET formation extracellular projection is seen on the hematoxylin stain (top, brightfield, grayscale). Sytox green highlights DNA projection from the center of the NET (bottom). (**B**)**.** At 3 h, thin DNA projection as well as center of the NET are visualized with Sytox green stain. Scale bar = 10 μm.

**Figure 7:**
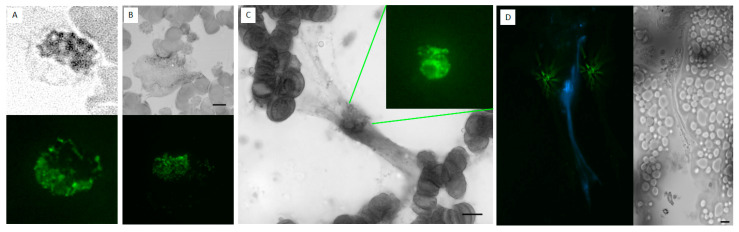
**MPO staining of PMA induced NETs.** EDTA-whole blood samples stimulated with PMA to induce NETs were stained with hematoxylin dye (brightfield, grayscale images) and MPO (fluorescent green images) at 45 min, 2 h, and 3.5 h. (**A**) At 45 min, MPO is confined within the neutrophil. (**B**) At 2 h NET formation is visible under hematoxylin stain and MPO is seen outside of cellular bounds. (**C**) At 3.5 h, on hematoxylin stain, mature NET is visualized with DNA projections extending from the center which stains strongly for MPO. (**D**) At 3.5 h MPO (fluorescent green) is seen located centrally and extending peripherally within the NET DNA projections positive for DAPI stain (fluorescent blue). Discoid, round cells in the periphery and near the scale bars are red blood cells, some forming rouleaux due to the fixation and smearing. Scale bar = 10 μm.

**Figure 8:**
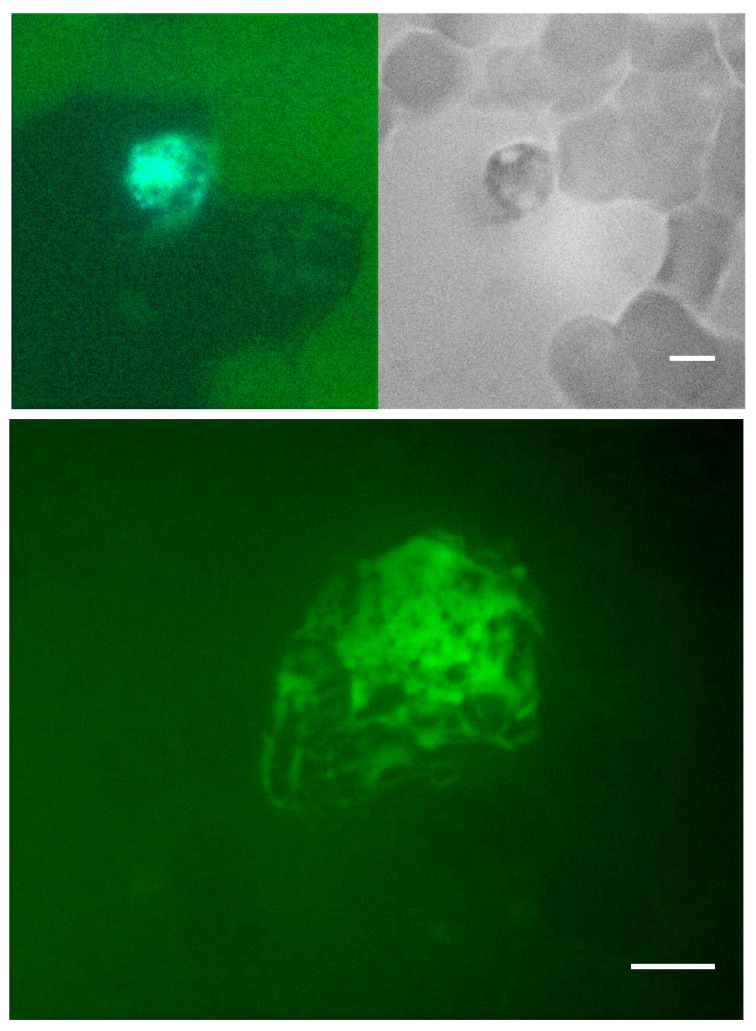
**Citrullinated Histone3 staining of PMA induced NETs.** EDTA-whole blood stimulated with PMA stained for citrullinated histone. Scale bar = 10 μm.

PMA, LPS, and Ionomycin were used to determine whether NETs could be induced in routinely collected EDTA-whole blood specimens (Figure 5). In samples incubated with PMA, LPS, and Ionomycin up to 24 h, we observed vacuolation within the leukocyte cytoplasm, followed by congestion and decondensation of the nuclei. Neutrophil degranulation and ejection of chromatin and nuclear material was observed as early as 30 min and continued for up to 8 h. After 6 h of incubation, most neutrophils converted into SC, morphologically resembling NETs. Further expansion of the extracellular nuclear material and chromatin was observed up to 24 h. After 24 h, many of the neutrophil remnants disappeared. Following the stimulation of whole blood with PMA, morphologically identified induced NETs stained strongly with Sytox green, MPO, and citrullinated histone H3 (Figure 6, Figure 7 and Figure 8).

**Figure 9:**
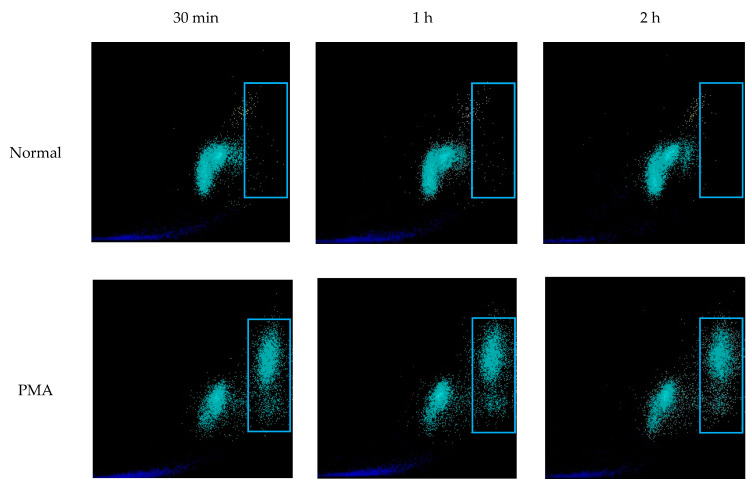
**WNR scattergrams of PMA stimulated EDTA-whole blood samples.** EDTA-whole blood samples from healthy donors were incubated with PMA to induce NETs. After 2 **h** of incubation the normal sample (no PMA added) did not have increased signal in the region of high nucleic acid content (SFL, blue rectangle). Blood samples stimulated with PMA developed progressively increasing signal at the region of high SFL. In the PMA-induced sample, as the intensity of signal in the high SFL region increased the signal for WBC cells decreased suggesting that a proportion of WBCs evolved into NETs and were now detected in high SFL region.

WNR scattergrams of PMA stimulated EDTA-whole blood samples (definitively containing NETs) demonstrated signal in the same area of WNR scattergram as seen in samples with ≥20% NET-like SC. These changes in the scattergram were observed as early as 30 min, and events in the large SFL area continuously increased up to two hours (Figure 9). This further affirms the identification of a NETs area in high SFL but moderate FSC on the WNR scattergram (Figure 9, blue rectangle).

### 3.8. % SC in EDTA Whole Blood Correlates with Presence of Infections

We reviewed the electronic medical records of 200 patients with ≤5% and 194 patients with ≥20% SC on CellaVision^®^. As shown in Table 1, 50.5% patients with ≥20% SC had infections, as compared to 31.5% patients with ≤5% SC, *p* < 0.001. This increase was true for both bacterial (*p* = 0.034) and viral infections (*p* < 0.001). The XN scattergrams of samples with ≥20% SC had a significantly higher signal in the area representing high nucleic acid content than scattergrams of samples with ≤5% SC, 1.6 [0.67, 3.3]) vs. 0.69 [0.29, 1.7], respectively (*p* < 0.001), suggesting the presence of NETs.

## 4. Discussion

NETs represent one of neutrophil’s defense mechanisms against septic and sterile inflammatory states [1,2,3,8,10]. They migrate to infected or inflamed tissues and have been identified within parenchyma of the affected organs [24]. Nevertheless, a quick, reliable, and clinically meaningful identification method for circulating NETs has not been developed. In addition to the use of immunohistochemistry for the direct visualization of NETs, studies also report using surrogate markers such as cell-free DNA, MPO-DNA complexes, citrullinated histones C3, neutrophil elastase, and cathelicidin antimicrobial peptides [13,14,15,16]. Additionally, the presence of serologic NET markers has been correlated to increased SFL signals on Sysmex WDF scattergrams in patients with both acute and chronic inflammatory states [18,20].

A possible explanation for the lack of reliable visualization techniques of circulating NETs is that they are difficult to identify without using NET specific stains and markers, which have not been integrated into clinical practices and are not readily available in strictly clinical settings. On digital images of peripheral smears generated by CellaVision^®^, at first glance, NETs resemble the classic smudged lymphocytes seen in peripheral smears of patients with CLL. Upon closer inspection, they differ from these lymphocytes in size and by their intricate network of fine extracellular projections. With the advent of digital imaging systems for morphological WBC differential and quantification, such as the CellaVision^®^, we observed that the SC category contains two distinct entities: degenerated lymphocytes and cell remnants resembling NETs. Such an observation opens up a new quick, easy, and readily available avenue for NET identification. To investigate this further, we developed a set of experiments to characterize these NET-like smudge cells.

Samples classified as normal by Sysmex had on average 4.3% SC. Typical lymphocyte smudge cells, as seen in patients with CLL, are a product of smear preparation, nevertheless we did not see a significant increase in NET-like SC when we altered specimen storage conditions and slide preparation technique. As described in prior studies, nucleic acid content of WBC represented by side scatter on WDF Sysmex scattergrams correlates with NET markers [18,20]. In our study, we used the WNR scattergram to show that samples with high contents of NET-like SC also had a unique signal that localized to the high SFL and moderate FSC area. Samples of patients with CLL, expected to have smudge cells, did not have this distinct pattern on the WNR scattergram. To further confirm our ability to differentiate between NETs and DL within the SC category we used LAP staining and flow cytometry. Both techniques confirmed that NET-like SC are of neutrophil origin. Samples with ≥20% NET-like SC stained strongly for LAP, MPO, and NE by immunohistochemistry and on flow cytometry were positive for surface DNA, CD45, MPO, and CD66b which is a marker of neutrophil activation that is upregulated during NETosis [24].

Identification of NETs is further complicated by their dynamic morphology. On digital peripheral smears of CellaVision^®^ we observed many morphologically distinct forms of NET-like SC. We hypothesize that these might represent different stages of NET formation and maturation. To test this, we developed an in-vitro model to study the morphological changes of neutrophils during NET formation using PMA, LPS, and Ionomycin. Using these triggers, we observed changes in neutrophils including vacuolation, nuclear decompensation and development of spider-web like projections in as early as 30 min. These changes were captured by the CellaVision^®^ and appeared similar to some of the NET-like SC seen in unmanipulated EDTA whole blood samples. NETs induced in peripheral blood samples with PMA, LPS, and Ionomycin resulted in a signal similar on the WNR scattergram to that produced by samples with ≥20% NET-like SC.

To apply our findings clinically, we correlated % SC with the presence of documented bacterial and viral infections in hospitalized patients. When compared to patients with ≤5% SC, those with ≥20% SC have a significantly higher incidence of infections within 10 days of specimen collection. WNR scattergrams of samples with ≥20% SC display a strong signal in the high SFL area of the WNR scattergram—a pattern unique to samples induced with NET triggers. This suggests that patients with high %SC identified by CellaVision^®^ may have an underlying inflammatory process driving NETosis. This study was limited to studying the correlation between % NET-like SC and infections and the sample size was not sufficiently powered to study the association of NET-like SC in other non-infectious diseases and conditions. Nonetheless, NETosis has been associated with many non-infectious conditions including cancer, thrombosis, autoimmune disorders, and chronic metabolic disorders [8,25,26,27,28,29]. Future studies are needed to establish the association of NET-like SC with these conditions. With the availability of automated and digitized blood cell differential count becoming routine not only in hospitals but also in community-based clinics, a pragmatic, fast, and cost-effective screen tool such as the one described herein has potential for wide applications and the early detection of NETosis in multiple diseases and disorders.

## 5. Conclusions

In this article, we propose that the smudge cell category of CellaVision^®^ likely contains two distinct populations of leukocytes: degenerated lymphocytes and NETs. NET-like smudge cells are not an artifact of smear preparation, are identified in the high SFL area of the WNR scattergram, and express neutrophil markers identifiable on immunohistochemistry and flow cytometry. In hospitalized patients, the percentage of smudge cells correlated with infections. The identification of NETs on digital images of peripheral blood smears generated by routinely used automated hematology systems such as CellaVision^®^ offers an easy, fast, cost-effective approach with a variety of possible clinical applications and potentially an early marker for infections.

## 6. Patents

Identifying neutrophil extracellular traps in biological samples: US20220254015A1, Publication Date: 11 August 2022.

## Figures and Tables

**Table 1:** Infection rates in samples with <5% SC and >20% SC.

	Control (≤5% SC) (n = 200)	High SC (≥20% SC) (n = 194)	*p*
Female, n (%)	108 (54.0)	103 (53.1)	0.94
Age, median [IQR]	59.9 [46.1, 69.7]	59.3 [41.1, 70.7]	0.64
% Smudge Cells (SC), median [IQR] WBC × 10^9^/L, median [IQR] XN Scattergram NETs area, median [IQR]	2.6 [1.7, 3.5] 8.7 [4.60, 14.7] 0.69 [0.29, 1.7]	33.0 [24.5, 45.7] 7.3 [3.7, 14.3] 1.6 [0.67, 3.3]	0.14 <0.001
Patients with infections *, n (%)	63 (31.5)	98 (50.5)	<0.001
Bacterial infections, n (%)	44 (22.0)	62 (32.0)	0.034
Viral infections, excluding HIV, n (%)	20 (10.0)	51 (26.3)	<0.001
Acute viral infection **, n (%)	15 (7.5)	29 (14.9)	0.029
Chronic viral infection ***, n (%)	7 (3.5)	22 (11.3)	0.005
HIV ****, n (%)	10 (5.0)	14 (7.2)	0.48

* Each patient could have more than one infection, ** excluding HIV, EBV, CMV, HCV, HBV, *** CMV, EBV, HCV, HBV, **** HIV not included in total number of infections.

## Data Availability

The data presented in this study are available on request from the corresponding author.
